# Short-Chain Fatty Acids Alleviate Hepatocyte Apoptosis Induced by Gut-Derived Protein-Bound Uremic Toxins

**DOI:** 10.3389/fnut.2021.756730

**Published:** 2021-10-12

**Authors:** Mingjuan Deng, Xingqi Li, Weiwei Li, Jiahui Gong, Xiaoying Zhang, Shaoyang Ge, Liang Zhao

**Affiliations:** ^1^Key Laboratory of Functional Dairy, College of Food Science and Nutritional Engineering, China Agricultural University, Beijing, China; ^2^Inner Mongolia Dairy Technology Research Institute Co., Ltd., Hohhot, China; ^3^Hebei Engineering Research Center of Animal Product, Sanhe, China; ^4^Department of Nutrition and Health, Beijing Advanced Innovation Center for Food Nutrition and Human Health, China Agricultural University, Beijing, China

**Keywords:** uremic toxins, hepatocytes, apoptosis, hippuric acid, short chain fatty acid

## Abstract

Chronic kidney disease (CKD) is characterized with the influx of uremic toxins, which impairs the gut microbiome by decreasing beneficial bacteria that produce short-chain fatty acids (SCFAs) and increasing harmful bacteria that produce gut-derived protein-bound uremic toxins (PBUTs). This study aimed to assess the proapoptotic effects of three major gut-derived PBUTs in hepatocytes, and the effects of SCFAs on apoptosis phenotype *in vitro*. HepG2 (human liver carcinoma cells) and THLE-2 (immortalized human normal liver cells) cell line were incubated with 0, 2, 20, 200, 2000 μM p-cresol sulfate (PCS), indoxyl sulfate (IS), and hippuric acid (HA), respectively, for 24 h. Flow cytometry analysis indicated that three uremic toxins induced varying degrees of apoptosis in hepatocytes and HA represented the highest efficacy. These phenotypes were further confirmed by western blot of apoptosis protein expression [Caspase-3, Caspase-9, B-cell lymphoma 2 (Bcl-2), and Bcl-2-associated X protein (Bax)]. Human normal hepatocytes (THLE-2) are more sensitive to PBUTs-induced apoptosis compared with human hepatoma cells (HepG2). Mechanistically, extracellular HA could enter hepatocytes, increase reactive oxygen species (ROS) generation, and decrease mitochondrial membrane potential dose-dependently in THLE-2 cells. Notably, coculture with SCFAs (acetate, propionate, butyrate) for 24 h significantly improved HA-induced apoptosis in THLE-2 cells, and propionate (500 μM) represented the highest efficacy. Propionate reduction of apoptosis was associated with improving mitochondria dysfunction and oxidative stress in a manner involving reducing Caspase-3 expression, ROS production, and increasing the Bcl-2/Bax level. As such, our studies validated PBUTs accumulation might be an important cause of liver dysfunction in patients with CKD, and supplementation of SCFAs might be a viable way to protect the liver for patients with CKD.

## Introduction

Chronic kidney disease (CKD) has become a serious health issue affecting 10–15% of the global population (1). The pathogenesis of CKD is characterized by kidney filtration dysfunction, leading to blood retention of uremic toxins (2). More than 130 uremic toxins have now been identified and categorized as small water-soluble molecules, middle-sized molecules, and protein-bound uremic toxins (PBUTs) (2, 3). Several of the most harmful PBUTs, including indoxyl sulfate (IS), p-cresol sulfate (PCS), and hippuric acid (HA), are produced by gut bacteria and are hard to be removed by current conventional dialysis (2, 4). Intestinal flora metabolizes tryptophan and tyrosine, leading to the production of indole and p-cresol, which are subsequently metabolized as PBUTs by hepatic cytosolic enzymes and finally secreted to the blood circulation (5–7). The accumulation of PBUTs could exert harmful biological activity on other tissues or organs, mainly on the kidney and the cardiovascular system (8, 9). Notably, patients with primary renal disease often have coexisting liver disease, which presents diagnostic and treatment challenges (10). Zhu et al. found that PCS induced oxidative stress, glutathione depletion, and cellular necrosis in human hepatocytes (11). IS and PCS could affect hepatic bile acid transport and mitochondrial functions (6, 8). In this respect, Weigand et al. revealed that HA mediated liver injury primarily *via* mitochondrial toxicity (12). These studies evidenced that PBUTs also induced liver injury, although the mechanism is still unclear. Apoptosis is critically involved in PBUTs-induced harmful biological activities. PCS induced adipocyte and cardiomyocyte apoptosis *via* mitochondria-related pathways (13, 14). Lin et al. found IS caused apoptosis *via* oxidative stress and mitogen-activated protein kinase (MAPK) signaling inhibition in human astrocytes (15). IS also triggered apoptosis in the myoblast resulting in uremic sarcopenia (16). However, scarce research has been conducted to explore the proapoptotic effects of PBUTS on the liver, an extremely important organ to the metabolism, which attracted our scientific interest.

Biochemical disorders in CKD have been confirmed to some degree as a consequence of insufficient short-chain fatty acids (SCFAs) production due to gut dysbiosis (17). SCFAs are the main intestinal bacterial metabolites of dietary fiber (18). The most abundant SCFAs in the human gut are acetate, propionate, and butyrate, which constitute over 95% of the total SCFAs content (18, 19). Previous evidence showed that SCFAs regulated metabolic processes as substrates and signaling molecules by entering the systematic circulation (20). Additionally, Hu et al. found that acetate and butyrate improved β-cell apoptosis-involved mitochondrial-dependent pathways (21). Furthermore, propionate was established to inhibit the apoptosis of human islet and hippocampus cells *via* mitochondrial dysfunction attenuation (22–24). Dietary supplementation with sodium butyrate alleviated dairy goat hepatocyte apoptosis (25). These findings implied the potential of SCFAs apoptosis pathway regulation. However, insufficient investigations have been performed so far to assess the effects of SCFAs on human hepatocyte apoptosis.

In this study, we explored the proapoptotic effect of three important gut-derived uremic toxins (HA, IS, and PCS) and their potential mechanisms of action on human hepatoma and hepatocyte cells. Furthermore, we also evaluated the impact of three major SCFAs (acetate, propionate, and butyrate) on hepatocyte apoptosis-related phenotypes.

## Materials and Methods

### Cell Culture

HepG2 cells, a human hepatoma cell line, were purchased from the Stem Cell Bank, Chinese Academy of Sciences (Shanghai, China), and cultured in Dulbecco's Modified Eagles Medium (DMEM, Gibco, USA) containing 10% fetal bovine serum (Gibco, USA) and 100 U/ml penicillin and streptomycin (Beyotime, China) in a 5% CO_2_ incubator at 37°C. THLE-2 cells, a human hepatocyte cell line, were purchased from the Stem Cell Bank, Chinese Academy of Sciences (Shanghai, China), and cultured in RPMI 1640 medium (Gibco, USA) containing 10% fetal bovine serum (Gibco, USA) and 100 U/ml penicillin and streptomycin (Beyotime, China) in a 5% CO_2_ incubator at 37°C.

### Cell Cytotoxicity Assays

An enhanced Cell Counting Kit-8 assay (Beyotime, China) was used to determine cell viability. HepG2 and THLE-2 cells were seeded at a density of 5 × 10^3^ per well onto flat-bottom 96-well culture plates (Corning, USA) and treated for 24 h with 0–5 mM PCS, IS, HA, potassium chloride (KCL) (Sigma, USA), or uremic toxin mixtures, respectively. The doses applied were set based on clinical uremic concentration data in patients with CKD. The following mean concentrations were used: 1,000 μM HA, 200 μM IS, and 100 μM PCS. The highest concentrations utilized were as follows: 2,000 μM HA, 1,000 μM IS, and 200 μM PCS (26, 27). KCL was used as a control treatment to exclude osmotic pressure interference since IS and PCS exist in the form of potassium salt in the circulation (4). Uremic toxins mixtures were admixed with the mean (Mixtoxins-L) and highest dose (Mixtoxins-H) of the three uremic toxins, respectively. The absorbance values of viable cells were finally determined at 450 nm using a microplate spectrophotometer (BioTek Winooski, VT, USA). The cell inhibitory rates were measured according to the product protocol.

### Hepatocyte Apoptosis Assessment

Annexin V-Fluorescein isothiocyanate/propidium iodide (FITC/PI) detection kit (Beyotime, China) was used for apoptosis assessment. To explore the proapoptotic effects of gut-derived PBUTs on hepatocytes, HepG2 and THLE-2 cells were seeded into six-well plates at a density of 1 x 10^6^ cells. The cells were treated for 24 h with 2, 20, 200, and 2,000 μM IS, PCS, and HA, correspondingly. To explore the effects of SCFAs on HA-induced apoptosis in hepatocytes, THLE-2 cells grown in a six-well plate were treated with 2,000 μM HA in the presence/absence of SCFAs (500 or 5,000 μM sodium acetate, 50 or 500 μM sodium propionate, and 50 or 500 μM sodium butyrate) (28) when the confluency reached 70–80%. After the treatment, the cells were washed with PBS, trypsinized, and resuspended in 1× binding buffer. Then, the cells were stained with 5 ul of annexin V-FITC and 10 uL of PI in the dark for 20 min at room temperature. The samples were analyzed by flow cytometry (Beckman Coulter Inc., Brea, CA, USA) after staining. Annexin V–/PI– indicated viable cells, Annexin V+/PI– early-stage apoptotic cells, Annexin V+/PI+ end-stage apoptotic cells, and Annexin V–/PI+ cell debris.

### Mitochondrial Membrane Potential Measurement

A mitochondrial membrane potential (MMP) assay kit with 5,5′,6,6′-tetrachloro-1,1′,3,3′-tetraethylbenzimidazolylcarbocyanine iodide (JC-1) (Beyotime, China) was used according to the product instruction. THLE-2 cells were seeded into a six-well plate and treated with various concentrations of HA for 24 h. The cells were next washed with PBS and incubated in JC-1 working solution for 20 min at 37°C in the dark. After the incubation, we discarded the supernatant and washed the cells with JC-1 dying buffer. A volume of 2 ml of complete DMEM medium further was added in each well of the six-well plate. JC-1 monomer (green fluorescence distribution with an excitation wavelength of 490 nm and emission wavelength of 530 nm) and j-aggregates (red fluorescence distribution with an excitation wavelength of 525 nm and emission wavelength of 590 nm) were measured by a fluorescent microscope (Olympus, Tokyo, Japan). Mitochondrial depolarization was indicated by the decrease in the red/green fluorescence intensity ratio.

### Intracellular Reactive Oxygen Species Production Measurement

A reactive oxygen species (ROS) assay kit (Beyotime, China) was used for intracellular ROS detection following the product guidelines. THLE-2 cells were seeded into a six-well plate and treated for 24 h with various concentrations of HA, PCS, and IS in the presence/absence of 500 μM sodium propionate. Cells were incubated with 2′, 7′-dichlorofluorescein diacetate (DCFH-DA) dissolved in serum-free DMEM (1:1,000) in the dark for 20 min. Fluorescence intensity was measured by flow cytometry and/or fluorescent microscope (Olympus, Japan) quantified by Image J software.

### Intracellular and Extracellular HA Determination

Ultra-high-performance liquid chromatography (HPLC, Waters, USA) coupled with a TripleTOF® 5600 plus (Applied Biosystems, Foster City, CA, USA) mass spectrometer was used to measure the intracellular and extracellular HA content in THLE-2 cells as previously described (29) with a modification in the sample preparation, which is detailed below. Cells were seeded into six-well plates at a density of 1 x 10^6^ cells. The cells were then treated with various doses of HA for 24 h or 15 min after confluency had reached 80%. After the treatment, the medium was collected and centrifuged at 350 g for 5 min. The pellet was discarded, and the supernatant was collected for extracellular HA measurement. The cells in the plates after the treatment were washed with PBS two times, scraped, and homogenized, and then centrifuged for 10 min at 4,000 g and 4°C. Finally, the supernatant was collected for intracellular HA determination.

### Western Blot Analysis

HepG2 and THLE-2 cells were cultured in a six-well plate and treated as described above. Cultured cells were prepared for Western blot analysis as reported previously (28). The membranes were incubated with primary antibodies: glyceraldehyde-3-phosphate dehydrogenase (GAPDH), B-cell lymphoma 2 (Bcl-2), Bcl-2-associated X protein (Bax), cleaved caspase-3, and cleaved caspase-9 (CST, USA). After the incubation with a goat anti-rabbit horseradish peroxidase-conjugated secondary antibody (Beyotime, China) at a dilution of 1:10,000 for 1 h, the proteins were visualized using a Luminata Forte Enhanced Chemiluminescence Kit (Millipore, Bedford, MA, USA), and the band intensities were analyzed using the QuantityOne analysis software (Bio-Rad, Hercules, CA, USA).

### Statistical Analysis

Numeric data were expressed as mean ± SD. Statistical significance was determined using the one-way ANOVA followed by least significant difference (LSD) posttests with statistical package for the social sciences (SPSS) 25.0. Differences were considered statistically significant at a two-tailed *p-*value < 0.05.

## Results

### Gut-Derived PBUTs Induced Apoptosis in HepG2 Cells

**Figure 1:**
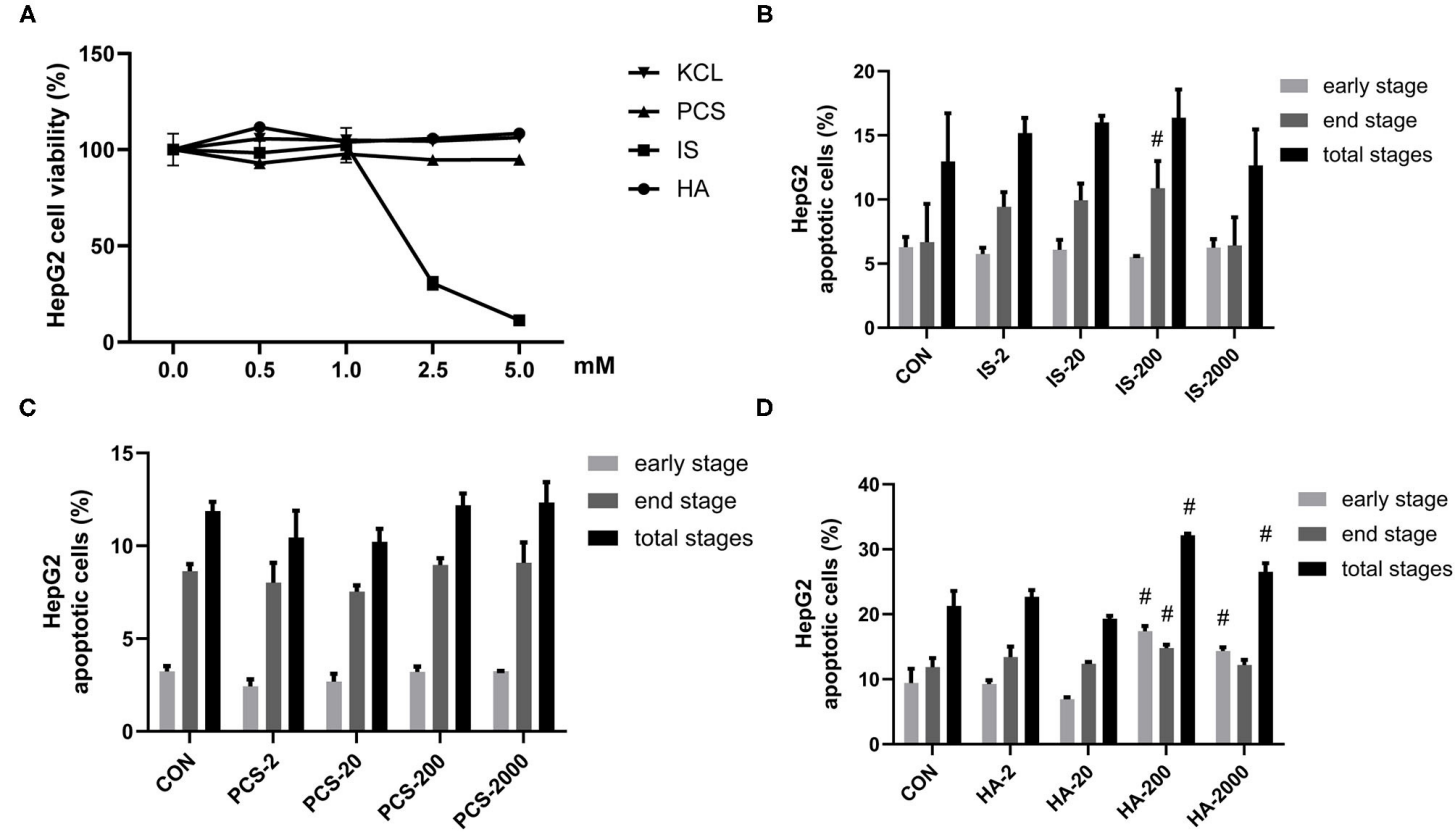
Gut-derived protein-bound uremic toxins (PBUTs) induced apoptosis in HepG2 cells. **(A)** PBUTs cytotoxic effects. CCK8 assay was performed for HepG2 cells treated with p-cresol sulfate (PCS), indoxyl sulfate (IS), hippuric acid (HA), and KCL at doses of 0-5 mM for 24 h (*n* = 3). **(B)** Effect of IS on HepG2 apoptosis (*n* = 3). After 24 h treatment, flow cytometry was performed on cells stained with annexin V-FITC and PI. CON, control. IS-2, 2 μM IS treatment. IS-20, 20 μM IS treatment. IS-200, 200 μM IS treatment. IS-2000, 2000 μM IS treatment. Group settings are the same with PCS **(C)** and HA **(D)**. **(C)** Effect of PCS on HepG2 apoptosis (*n* = 3). **(D)** Effect of HA on HepG2 apoptosis (*n* = 3). For all bar graphs, the data are the mean ± SD. ^#^, *P* < 0. 05 vs. CON group. The significant difference was assessed using the one-way ANOVA followed by LSD posttests.

Uremic syndrome coupled with kidney dysfunction is characterized by retention of uremic toxins, and several of the most harmful uremic toxins are produced by gut bacteria, including HA, IS, and PCS (5). These toxins are protein-bound and are, therefore, hard to remove, which leads to their abnormal blood circulating concentrations, exert harmful biological activity on other organs (27). Although PCS was found to induce oxidative stress, glutathione depletion, and cellular necrosis in a human liver cell line, more evidence is needed to clarify the effects of gut-derived PBUTs on hepatocytes (11). In this study, we determined the effects of 0–5 mM HA, IS, PCS, and KCL on HepG2 cell viability (Figure 1A). Cell viability was not significantly reduced by the incubation with 0–5 mM PCS or HA for 24 h. The KCL treatment failed to influence HepG2 cell viability, which excluded the potential interference of the potassium salt form of IS and PCS on the osmotic pressure. The treatment with 0–1 mM IS for 24 h did not alter HepG2 cell viability, whereas doses beyond 1 mM caused a significant reduction in cell viability (Figure 1A).

Next, we sought to examine the proapoptotic effects of three PBUTs in HepG2 cells. Notably, the treatment with 200 μM IS for 24 h markedly elevated the end-stage apoptosis with no significant alterations achieved in the other treatments (Figure 1B; Supplementary Figure S1A). As for PCS, various doses incubation for 24 h failed to induce apoptosis in HepG2 cells (Figure 1C; Supplementary Figure S1B). Notably, we found that the HA treatment with 200 and 2,000 μM in of the HepG2 cells led to a significantly higher total apoptosis cell percentage than that of the control group, by 10.86% and 5.26% (Figure 1D; Supplementary Figure S1C).

### Gut-Derived PBUTs Induced Apoptosis in the THLE-2 Cells

**Figure 2:**
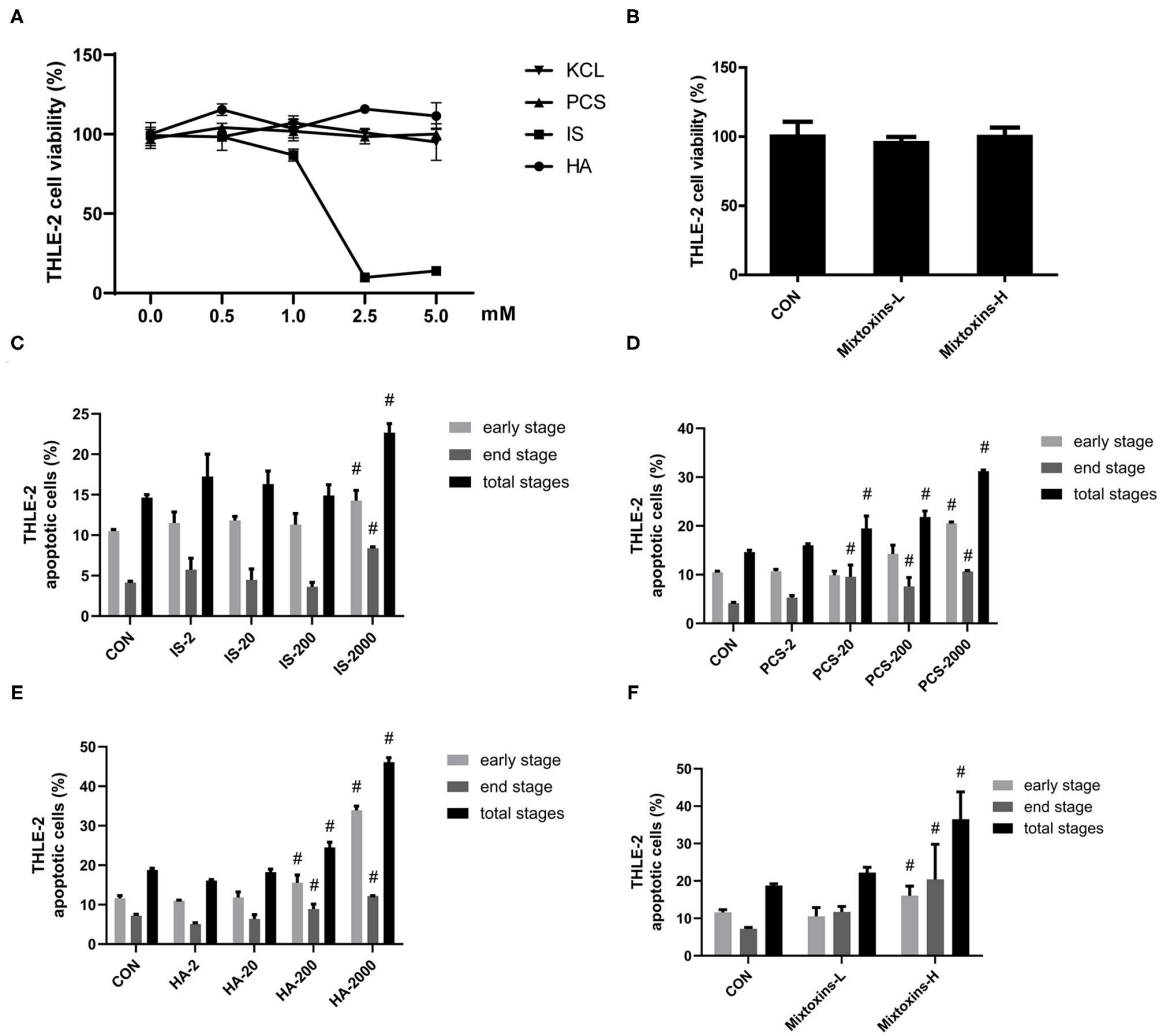
Gut-derived PBUTs induced apoptosis in THLE-2 cells. **(A)** PBUTs cytotoxic effects. CCK8 assay was performed for THLE-2 cells treated with PCS, IS, HA, and KCL at doses of 0-5 mM for 24 h (*n* = 3). **(B)** Effects of three PBUTs mixture on THLE-2 cell viability (*n* = 5). **(C)** Effect of IS on THLE-2 apoptosis (*n* = 2-3). After 24 h treatment, flow cytometry was performed on cells stained with annexin V-FITC and PI. CON, control. IS-2, 2 μM IS treatment. IS-20, 20 μM IS treatment. IS-200, 200 μM IS treatment. IS-2000, 2000 μM IS treatment. Group settings are the same with PCS **(D)** and HA **(E)**. **(D)** Effect of PCS on THLE-2 apoptosis (*n* = 2-3). **(E)** Effect of HA on THLE-2 apoptosis (*n* = 3). **(F)** Effects of three gut-derived PBUTs mixture on THLE-2 apoptosis (*n* = 3). For all bar graphs, the data are the mean ± SD. #, *P* < 0. 05 vs. CON group. The significant difference was assessed using the one-way ANOVA followed by LSD posttests.

We also examined the metabolic effects of IS, PCS, and HA on the human hepatocyte cell line THLE-2. The treatment and concentrations were set as detailed in section Materials and Methods. Similarly, to the results we obtained in the HepG2 cells, the THLE-2 cell viability was not significantly reduced by incubation with 0–5 mM PCS, HA and 0–1 mM IS for 24 h, whereas IS beyond 1 mM markedly decreased THLE-2 cell viability (Figure 2A). PBUTs are always present together in the system circulation of patients with CKD (30), which raised our research interest in the effects of PBUTs mixture on hepatocytes. We found that neither the low (HA: 1,000 μM, IS: 200 μM, PCS: 100 μM), nor the high doses (HA: 2,000 μM, IS: 1,000 μM, PCS: 200 μM) of PBUTs mixture altered the THLE-2 cell viability as compared with that of the control group (Figure 2B).

The treatments with 2, 20, and 200 μM IS for 24 h failed to induce apoptosis in THLE-2 cells, whereas the treatment with 2,000 μM IS induced 0.36-, 1.02-, and 0.55-fold increases in the early, late, and total apoptosis percentages as compared with the control group (Figure 2C; Supplementary Figure S2A). The incubation of 2,000 μM PCS with THLE-2 cells for 24 h induced significantly higher apoptosis, 0.96, 1.57, and 1.13-fold in the early, late, and total apoptosis, as compared with the control group (Figure 2D; Supplementary Figure S2B). The treatments with 200 and 2,000 μM HA induced significantly higher THLE-2 cell apoptosis in a dose-dependent manner. The incubation with 2,000 μM HA for 24 h resulted in increased early, late, and total apoptosis levels to 33.89, 12.17, and 46.07%, correspondingly, which was 1.92-, 0.69-, and 1.45-fold higher than the respective values in the control group (Figure 2E; Supplementary Figure S2C). The high doses of the three PBUT mixtures significantly increased the apoptosis level in the THLE-2 cells. The total apoptosis percentage was 17.69% higher followed by that of the late apoptosis (13.23%) and the early apoptosis (4.45%), as compared with the control group respective values (Figure 2F; Supplementary Figure S2D).

### Effects of PBUTs on the Apoptotic Protein Expression in Hepatocytes

**Figure 3:**
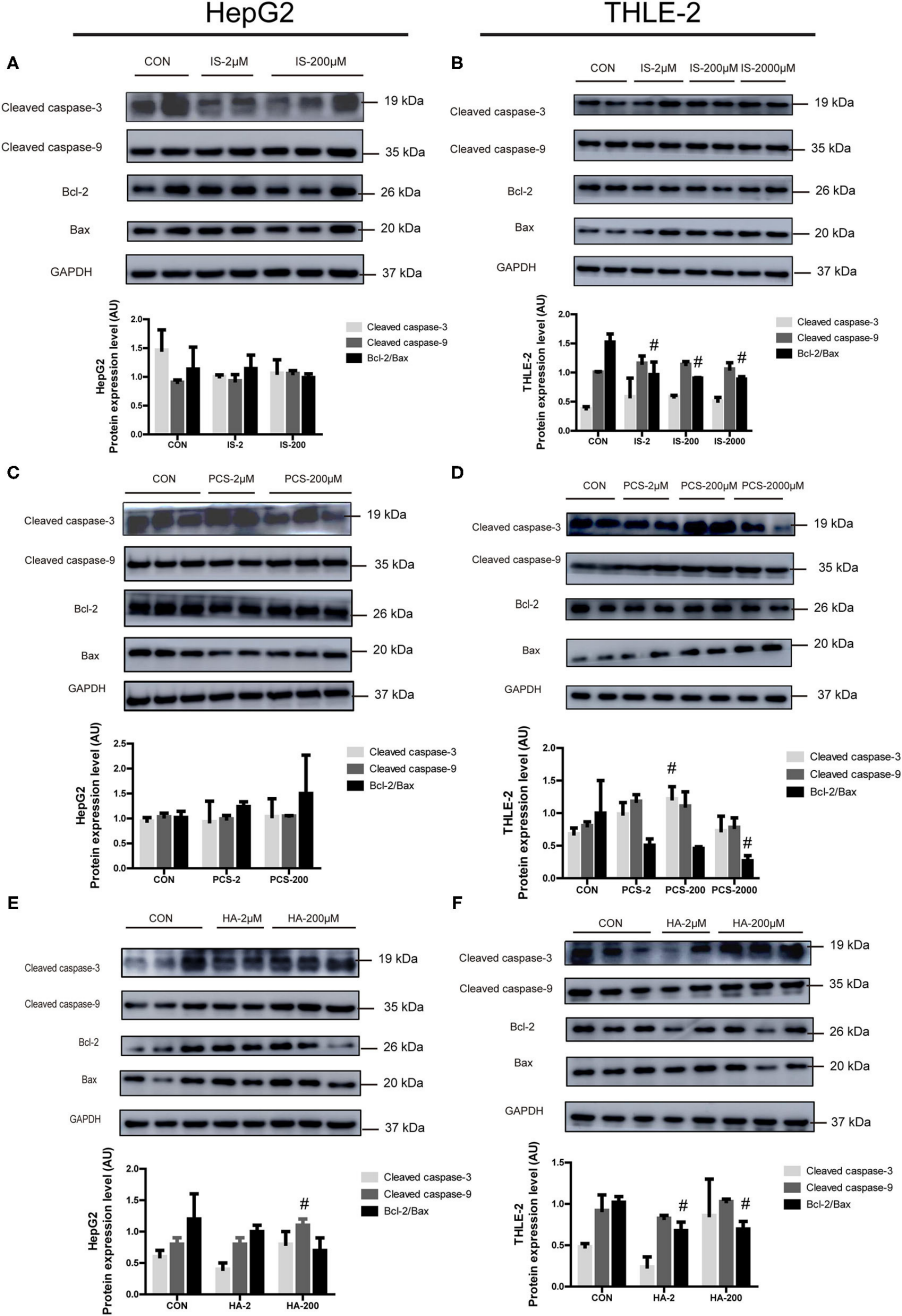
Effects of PBUTs on the apoptotic apoptosis protein expression in hepatocytes. HepG2 and THLE-2 cells were treated as detailed in section “Materials and Methods.” **(A,B)** Effects of IS on apoptosis protein expression. **(C,D)** Effects of PCS on apoptosis protein expression. **(E,F)** Effects of HA on apoptosis protein expression. HepG2 and THLE-2 cells were examined for apoptosis protein using Western blot. Bar graphs display quantification of blots. For all bar graphs, the data are the mean ± SD (*n* = 2-3). #, *P* < 0. 05 vs. CON group. The significant difference was assessed using the one-way ANOVA followed by LSD posttests.

We sought to examine the effects of the three PBUTs studied on the apoptosis signaling in hepatocytes by Western blot analysis, and determination of the levels of crucial apoptotic proteins, including cleaved caspase-3, cleaved caspase-9, Bcl-2, and Bax. The treatment of cultured HepG2 cells with 2, 200 μM IS for 24 h did not cause significant changes in the apoptotic protein expression (Figure 3A). In the THLE-2 cells, various doses of IS significantly decreased the Bcl-2/Bax expression level, which functions as an important apoptosis regulator (31) (Figure 3B). The treatments with 2 and 200 μM PCS did not alter significantly the apoptotic protein expression in HepG2 cells (Figure 3C). However, the treatment with 200 μM PCS increased the cleaved caspase-3 expression, but 2,000 μM PCS significantly decreased the Bcl-2/Bax expression level in the THLE-2 cells (Figure 3D). The treatment with 200 μM HA significantly elevated the cleaved caspase-9 expression in the HepG2 cells (Figure 3E); the incubation with both 2 and 200 μM HA for 24 h significantly decreased the Bcl-2/Bax expression level in the THLE-2 cells (Figure 3F). These results, together with the findings displayed in Figures 1, 2, strongly indicate that the three PBUTs induced partial apoptosis in the hepatocytes.

### PBUTs Induced ROS Generation and Mitochondrial Damage in the Hepatocytes

**Figure 4:**
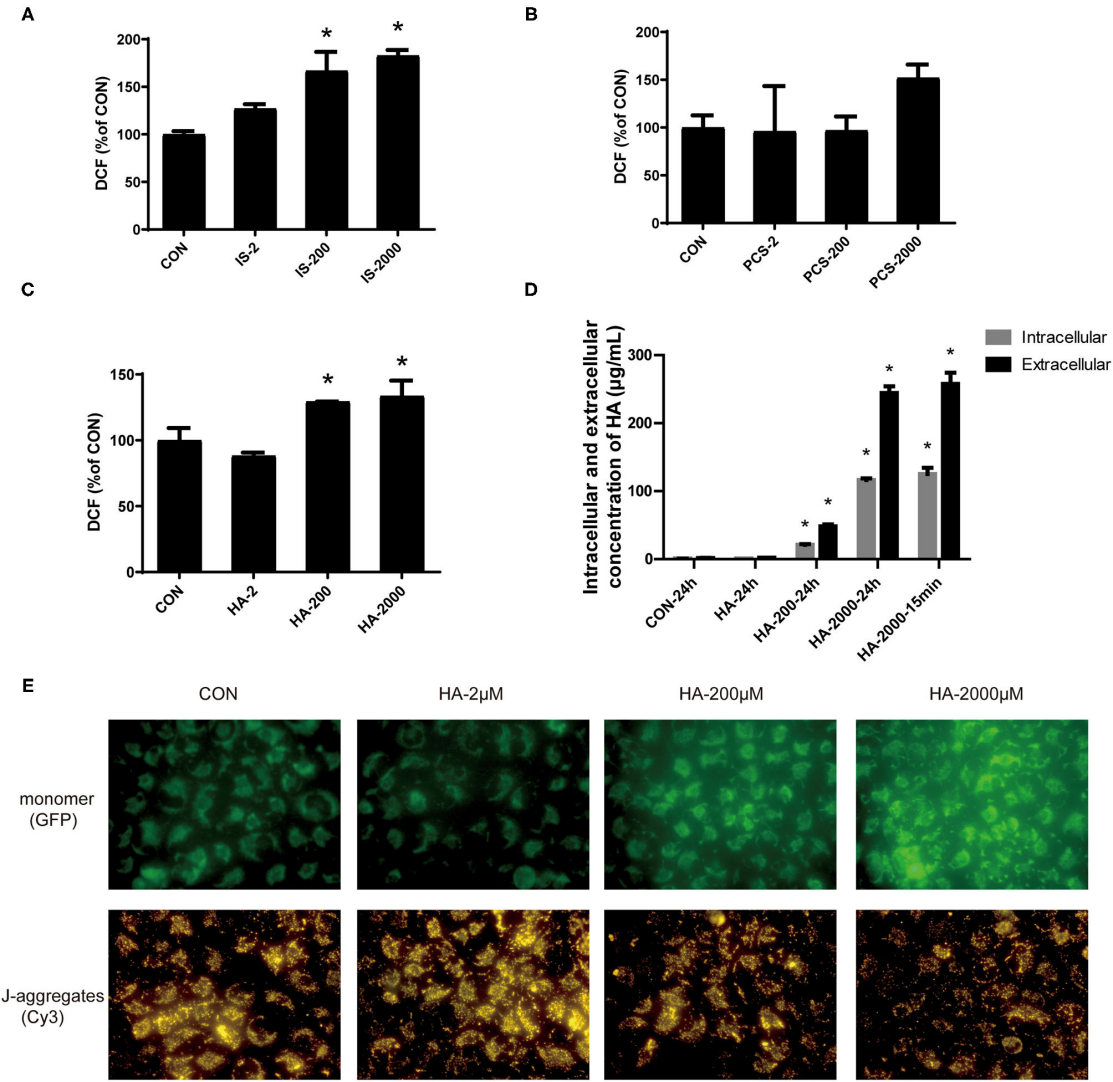
PBUTs induced reactive oxygen species (ROS) generation and mitochondria mitochondrial damage in the hepatocytes. **(A)** Effects of IS on ROS production in THLE-2 cells. **(B)** Effects of PCS on ROS production in THLE-2 cells. **(C)** Effects of HA on ROS production in THLE-2 cells. After treatment, cells were stained with DCFH-DA and measured by flow cytometry (*n* = 3). **(D)** Intracellular and extracellular HA concentration in THLE-2 cells cultured with different doses of HA after 24 h or 15 min (*n* = 3). **(E)** Effects of HA on mitochondrial membrane potential (MMP) in THLE-2 cells. After 24 h HA treatment, cells were stained with JC-1 solution to measure MMP level. For all bar graphs, data are the mean ± SD. *, *P* < 0. 05 vs. CON group. The significant difference was assessed using the one-way ANOVA followed by LSD posttests.

Reactive oxygen species have been tightly linked to the activation of the mitochondria-related apoptosis pathway (32). We measured the effects of gradient doses of PBUTs on the ROS production in THLE-2 cells. The IS or HA treatments with 200 and 2,000 μM for 24 h induced significantly higher ROS generation in the THLE-2 cells, whereas the PCS treatment exerted no significant alteration (Figures 4A–C). We chose HA for further investigation since HA had the highest proapoptotic efficacy among the three PBUTs based on our aforementioned results. Previously, HA had been proven to induce mitochondrial dysfunction in human endothelial cells (33), but the underlying mechanism is yet unknown. We aimed to elucidate whether HA could enter into the cells and act directly on their biological activity. Our results showed that 24 h of incubation with 200 or 2,000 μM HA dose-dependently increased the intracellular HA concentration in the THLE-2 cells (Figure 4D). Notably, we established that the incubation with 2,000 μM HA for 24 h or 15 min caused no significant difference in the intracellular HA concentration, which indicated that extracellular HA entered the hepatocytes and reached a dynamic balance within 15 min (Figure 4D). The incubation with 200 or 2000 μM HA for 24 h considerably increased the ROS production in a dose-dependent manner (Supplementary Figure S3). MMP decline is an important marker during cell apoptosis (34), and thus we also measured the alteration in MMP level induced in THLE-2 cells by the HA treatment. The diverse doses of HA induced obvious dose-dependent increases in the green fluorescence intensity and decreases in the red fluorescence as compared with the control (Figure 4E), which indicated that HA reduced the MMP level. These results revealed that HA might induce apoptosis involving ROS generation increase and mitochondrial damage.

### Propionate Alleviated HA-Induced Apoptosis in THLE-2 Cells

**Figure 5:**
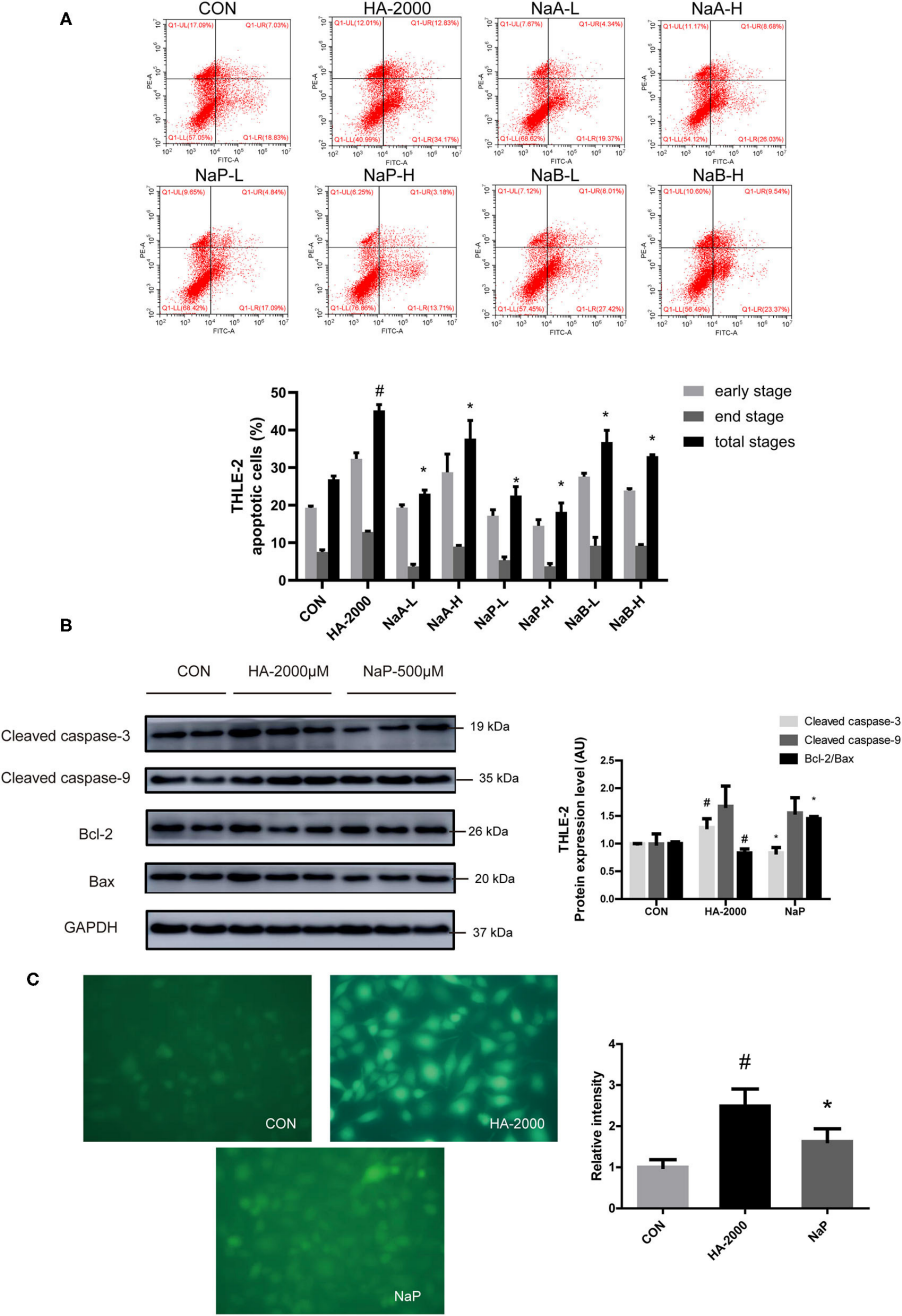
SCFA improved PBUT-induced apoptosis in THLE-2 cells. **(A)** Effects of SCFAs on HA-induced apoptosis in THLE-2 cells (*n* =3). CON, control group. HA-2000, 2000 μM. NaA-L, 500 μM sodium acetate. NaA-H, 5000 μM sodium acetate. NaP-L, 50 μM sodium propionate. NaP-H, 500 μM sodium propionate. NaB-L, 50 μM sodium butyrate. NaB-H, 500 μM sodium butyrate. **(B)** Propionate improved HA-induced apoptosis protein expression in THLE-2 cells (*n* = 2-3). **(C)** Propionate improved HA-induced ROS production in THLE-2 cells (*n* =3). For all bar graphs, the data are the mean ± SD. #, *P* < 0. 05 vs. CON group. *, *P* < 0. 05 vs. HA-2000 group. The significant difference was assessed using the one-way ANOVA followed by LSD posttests.

Short-chain fatty acids are the main microbial metabolites of dietary fibers in the gut that participate in metabolic processes as substrates or signaling molecules (19, 28). Recent studies showed that SCFAs improved β-cell apoptosis involved mitochondrial-related ROS suppression (21). In this study, we explored the effects of three major SCFAs (acetate, propionate, and butyrate) on HA-induced apoptosis in a human liver cell line (THLE-2). Surprisingly, we found that the coculture with various doses of acetate, propionate, and butyrate, respectively, for 24 h improved the apoptosis induced by the treatment with 2,000 μM HA (Figure 5A). Of the three SCFAs investigated, sodium propionate had the highest efficacy. The treatment with 500 μM (NaP-H) and 50 μM (NaP-L) propionate markedly reduced the apoptosis, which was 1.48-fold and 1.00-fold lower than that in the HA-2000 group. Then, we examined the effects of propionate on the apoptotic protein expression in the THLE-2 cells. Notably, 500 μM propionate cocultured with THLE-2 cell for 24 h reversed the increase in the cleaved caspase-3 and the decrease in the Bcl-2/Bax expression levels induced by the HA treatment (Figure 5B). Besides, the treatment with 500 μM propionate significantly reduced the ROS production induced by HA as well (Figure 5C). These results revealed that propionate improved the HA-induced hepatocyte apoptosis by decreasing ROS generation and caspase-3 protein expression, but increasing the Bcl-2/Bax protein expression level.

## Discussions

The accumulation of uremic toxins is the most prominent feature of CKD and end-stage renal disease. Uremic toxins are conventionally classified based on the physicochemical characteristics affecting their clearance during dialysis, of which PBUT is an important category. Several PBUTs originate from the gut and are metabolized in the liver (6), which have been confirmed to impair insulin resistance, kidney fibrosis, granulocyte function, and cardiovascular health (9, 35). Several recent studies evidenced that PBUTs induced oxidative stress, glutathione depletion, cellular necrosis, and bile acid transport disorders in the liver (6, 8, 11, 12). However, more evidence is needed to clarify the impact of PBUTs on the liver. The present study provides the primary data concerning the direct effects of three gut-derived PBUTs (HA, PCS, and IS) on human hepatocytes (THLE-2) and hepatoma cells (HepG2), focusing on the involvement of apoptotic pathways in which mitochondria are tightly involved.

Gut-derived PBUTs are bioactive microbiota metabolites originating exclusively from protein fermentation realized by the gut flora; they enter the blood circulation, where they are sulfated in the liver (36). Current clinical data indicated that the plasma level of PBUTs is rather low in the healthy population (HA: 16 μM, IS: 2 μM, PCS: 10 μM), but their average concentrations increase sharply in patients with CKD (HA: 398 μM, IS: 108 μM, PCS: 111 μM), as well as their highest concentrations (HA: 2,631 μM, IS: 940 μM, PCS: 219 μM) (27, 37). Existing evidence indicated that PBUTs induced cellular apoptosis in the kidneys, brain, muscles, bones, and the heart (14–16, 38, 39), whereas no such evidence for the liver. In the present study, IS and PCS exerted limited proapoptotic effects on HepG2 cells, whereas 2,000 μM IS and PCS induced marked apoptosis in the THLE-2 cells. HA (200 and 2,000 μM) induced apoptosis in both THLE-2 and HepG2 with the highest efficacy among the three PBUTs. The different apoptotic effects of the three PBUTs might be partly due to their involvement in diverse metabolic pathways in the liver. PCS and IS are sulfated in the hepatic Golgi apparatus and the endoplasmic reticulum (2, 40). HA is synthesized by the combination of benzyl-CoA and glycine in the mitochondrial matrix, which is more likely to induce apoptosis by damaging the mitochondria in toxic concentrations (26, 41). However, further investigation is needed to confirm this hypothesis. Our results also indicated that the THLE-2 cells were more sensitive to PBUTs-induced apoptosis than the HepG2 cells; all three PBUTs induced apoptosis in the THLE-2 cells, whereas only the high dose of HA exerted this effect in the HepG2 cells. This might be explained by the programmed cell death escape of the cancer cells by disordering apoptosis pathways (42, 43). The apoptotic effects of the three PBUTs mixtures on THLE-2 cells were also measured by the dose simulation of the average and highest plasma level in patients with CKD, whereas markedly induced apoptosis was observed only in the high-dose groups (HA: 2,000 μM, IS: 1,000 μM, and PCS: 200 μM). This finding provides further evidence for the positive feedback between PBUTs accumulation and liver injury in patients with CKD (44, 45).

Moreover, PBUTs treatment markedly increased ROS production, caspase-3 protein expression, but decreased the Bcl-2/Bax protein level in the THLE-2 cells. These proteins are considered to be mitochondria-mediated apoptosis regulators (46). Oxidative stress plays a crucial role in the potential apoptotic mechanism of PBUTs action. Supporting evidence exist that 500 μM PCS induced cardiac apoptosis partly *via* triggering NADPH oxidase activity and ROS production (14). Additionally, 10 μM IS induced apoptosis in human astrocytes through oxidative stress induction and MAPK pathway inhibition (15). Moreover, 40 mM HA triggered apoptosis mediated partly by p53 and endoplasmic reticulum stress in pig renal tubular cells (47). Notably, we found that extracellular HA entered hepatocytes reaching dynamic balance within 15 min, increasing the ROS production and reducing the MMP level, which provided the potential for HA to directly damage the mitochondria by entering the hepatocytes (12). HA is not eliminated by the liver, and avid influx and efflux occur across the basolateral membrane, mediated by the hepatic monocarboxylate transporter 2 (48, 49). Mitochondria could be the target of uremic toxins, and thus consequent mitochondrial damage might directly affect the production of uremic toxins; hence, a positive feedback circle emerges leading to the accumulation of uremic solutes, exerting harmful influence on the liver (3). Our present findings indicate that a toxic concentration of HA may enter the hepatocytes, causing mitochondrial damage and ROS production, and thus inducing hepatocyte apoptosis.

Short-chain fatty acids, predominantly acetate, propionate, and butyrate, are the main microbial metabolites of dietary fibers in the gut. Our previous studies confirmed that SCFAs improved hepatic dysfunction by modulating lipid metabolism and inflammation (28). The present study revealed that three major SCFAs improved HA-induced apoptosis in human hepatocytes, of which propionate had the highest antiapoptotic efficacy and was selected for research to obtain mechanistic insights. The mechanisms of the antiapoptotic effects of each SCFA might be different and network-like. Supporting evidence exist that at concentrations lower than 1 mM, acetate and butyrate inhibit apoptosis *via* improving mitochondrial dysfunction and ROS production, whereas higher concentrations have adverse effects on human islet cells (50). A concentration of 4 mM butyrate and propionate induced apoptosis not only in cancer cells but also in normal neutrophils by histone deacetylase (HDAC) inhibitor activity without the involvement of the G-protein-coupled receptor (GPR)-41/GPR-43 pathway (50). Acetate and propionate are confirmed to protect the islets from apoptosis in a GPR43-dependent manner through reduced caspase-3/7 activities (21, 22). Butyrate attenuated neuronal apoptosis *via* GPR41/Gβγ/PI3K/Akt pathway in rats (51). Recent studies showed that the mitochondrial function, GPRs, caspase-3 pathways, and HDAC activity might play a role in the antiapoptosis effects of SCFA. Furthermore, SCFA might exert positive impacts by enhancing the apoptosis of tumor cells and alleviating that of normal cells in various organs or tissues. Recently, reduced oxidative stress was found to account, at least partially, for propionate actions, because its effects, such as reduced caspase-3 expression, ROS production, but increased Bcl-2/Bax level, are tightly associated with mitochondrial-dependent apoptosis pathways (46). Although propionate was confirmed to inhibit apoptosis *via* increasing mitochondrial antioxidant enzyme levels and inducing autophagy to initiate mitochondria biogenesis (24, 52), further research is needed to elucidate the mechanism of action of propionate on the mitochondria.

In conclusion, the present study is the first to reveal that PBUTs (HA, PCS, and IS) induce apoptosis in human hepatocytes, of which HA has the highest efficacy. Normal liver cells (THLE-2) are more sensitive to PBUTs-induced apoptosis than liver cancer cells (HepG2). SCFAs (acetate, propionate, and butyrate) alleviate HA-induced apoptosis in THLE-2 cells, of which the treatment with propionate has the highest efficacy. Mechanistically, HA may induce liver apoptosis by entering hepatocytes, causing mitochondrial damage and ROS production. Propionate reduction of apoptosis is associated with mitochondrial dysfunction and oxidative stress improvement by reducing caspase-3 expression, ROS production, but by increasing the Bcl-2/Bax level. Therefore, our study confirms that PBUTs accumulation might be an important cause of liver dysfunction in patients with CKD. Notably, the supplementation of SCFAs might be a viable strategy for liver protection in patients with CKD.

## Data Availability Statement

The original contributions presented in the study are included in the article/Supplementary Material, further inquiries can be directed to the corresponding author/s.

## Author Contributions

MD, XL, WL, and JG carried out most experiments involving cells. MD and XL acquired data and analyzed data. XZ, SG, and LZ contributed to the scientific discussion. MD wrote the manuscript. LZ came up with the study concept and revised the manuscript. All authors contributed to the article and approved the submitted version.

## Funding

This study was supported by the National Natural Science Foundation of China (32072196) and the 111 Project from the Education Ministry of China (No. B18053).

## Conflict of Interest

XZ is employed by Inner Mongolia Dairy Technology Research Institute Co., Ltd. The remaining authors declare that the research was conducted in the absence of any commercial or financial relationships that could be construed as a potential conflict of interest.

## Publisher's Note

All claims expressed in this article are solely those of the authors and do not necessarily represent those of their affiliated organizations, or those of the publisher, the editors and the reviewers. Any product that may be evaluated in this article, or claim that may be made by its manufacturer, is not guaranteed or endorsed by the publisher.

## Supplementary Material

Supplementary Figure S1 **(A)** Effect of IS on HepG2 apoptosis (*n* = 3). After 24 h treatment, flow cytometry was performed on cells stained with annexin V-FITC and PI. CON, control. IS-2, 2 μM IS treatment. IS-20, 20 μM IS treatment. IS-200, 200 μM IS treatment. IS-2000, 2000 μM IS treatment. Group settings are the same with PCS **(B)** and HA **(C)**. **(B)** Effect of PCS on HepG2 apoptosis (*n* = 3). **(C)** Effect of HA on HepG2 apoptosis (*n* = 3).

Supplementary Figure S2 **(A)** Effect of IS on THLE-2 apoptosis (*n* = 2-3). After 24 h treatment, flow cytometry was performed on cells stained with annexin V-FITC and PI. CON, control. IS-2, 2 μM IS treatment. IS-20, 20 μM IS treatment. IS-200, 200 μM IS treatment. IS-2000, 2000 μM IS treatment. Group settings are the same with PCS **(B)** and HA **(C)**. **(B)** Effect of PCS on THLE-2 apoptosis (*n* = 2-3). **(C)** Effect of HA on THLE-2 apoptosis (*n* = 3). **(D)** Effects of three gut-derived PBUTs mixture on THLE-2 apoptosis (*n* = 3).

Supplementary Figure S3 Effects of HA-induced oxidative stress in THLE-2 cells. ROS level showed by fluorescent pictures after being treated with doses of HA, quantified by Image J software (*n* = 5). Data are the mean ± SD. ^*^, *P* < 0. 05 vs. CON group. The significant difference was assessed using the one-way ANOVA followed by LSD posttests.
