# Supplementary figures and images for: Short-Chain Fatty Acids Alleviate Hepatocyte Apoptosis Induced by Gut-Derived Protein-Bound Uremic Toxins
Source: Front Nutr. 2021 Oct 12;8:756730. doi: 10.3389/fnut.2021.756730 (PMC8545797; doi:10.3389/fnut.2021.756730)

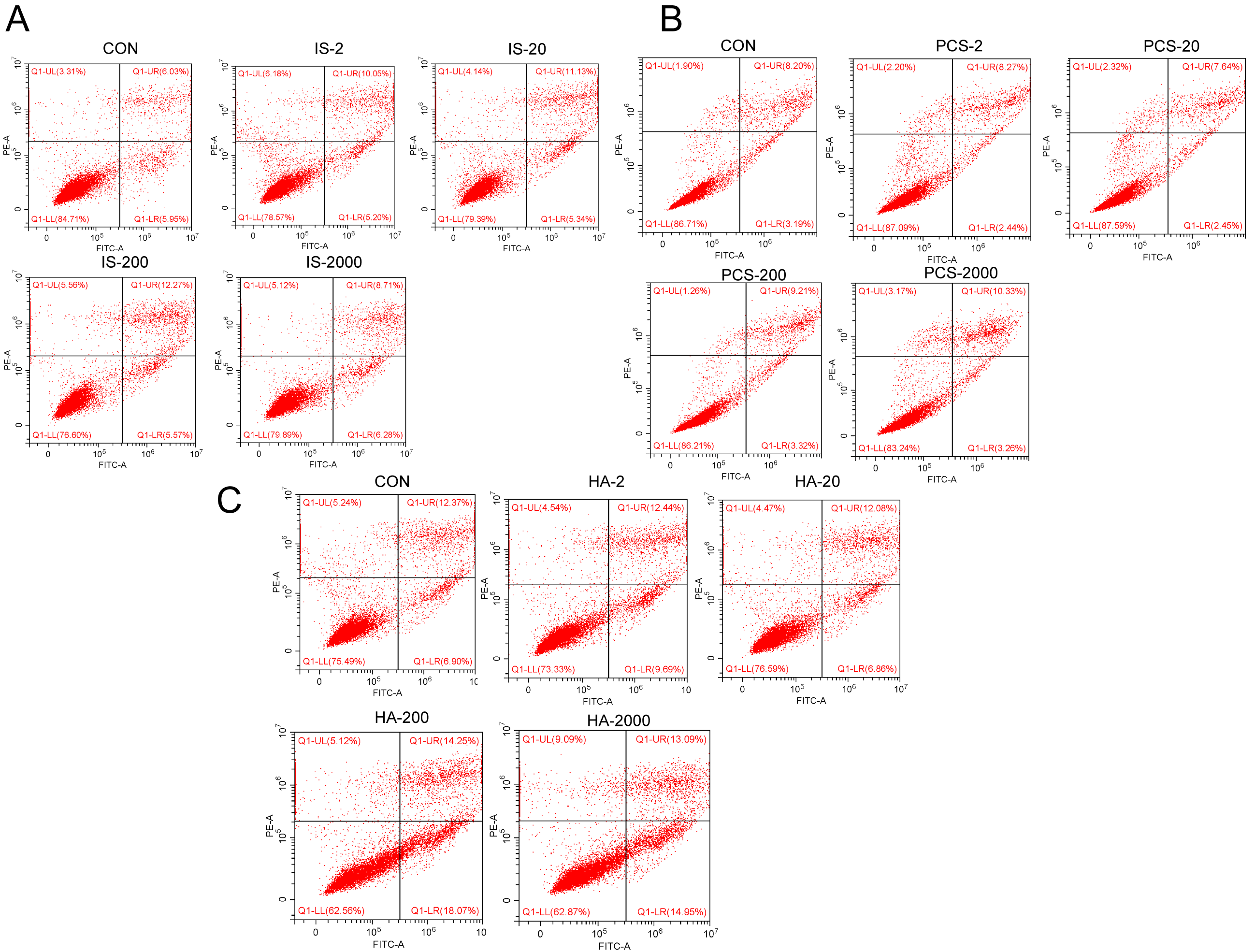

Supplement: Supplementary Figure S1 — (A) Effect of IS on HepG2 apoptosis (n = 3). After 24 h treatment, flow cytometry was performed on cells stained with annexin V-FITC and PI. CON, control. IS-2, 2 μM IS treatment. IS-20, 20 μM IS treatment. IS-200, 200 μM IS treatment. IS-2000, 2000 μM IS treatment. Group settings are the same with PCS (B) and HA (C). (B) Effect of PCS on HepG2 apoptosis (n = 3). (C) Effect of HA on HepG2 apoptosis (n = 3). [file Image_1.TIF]

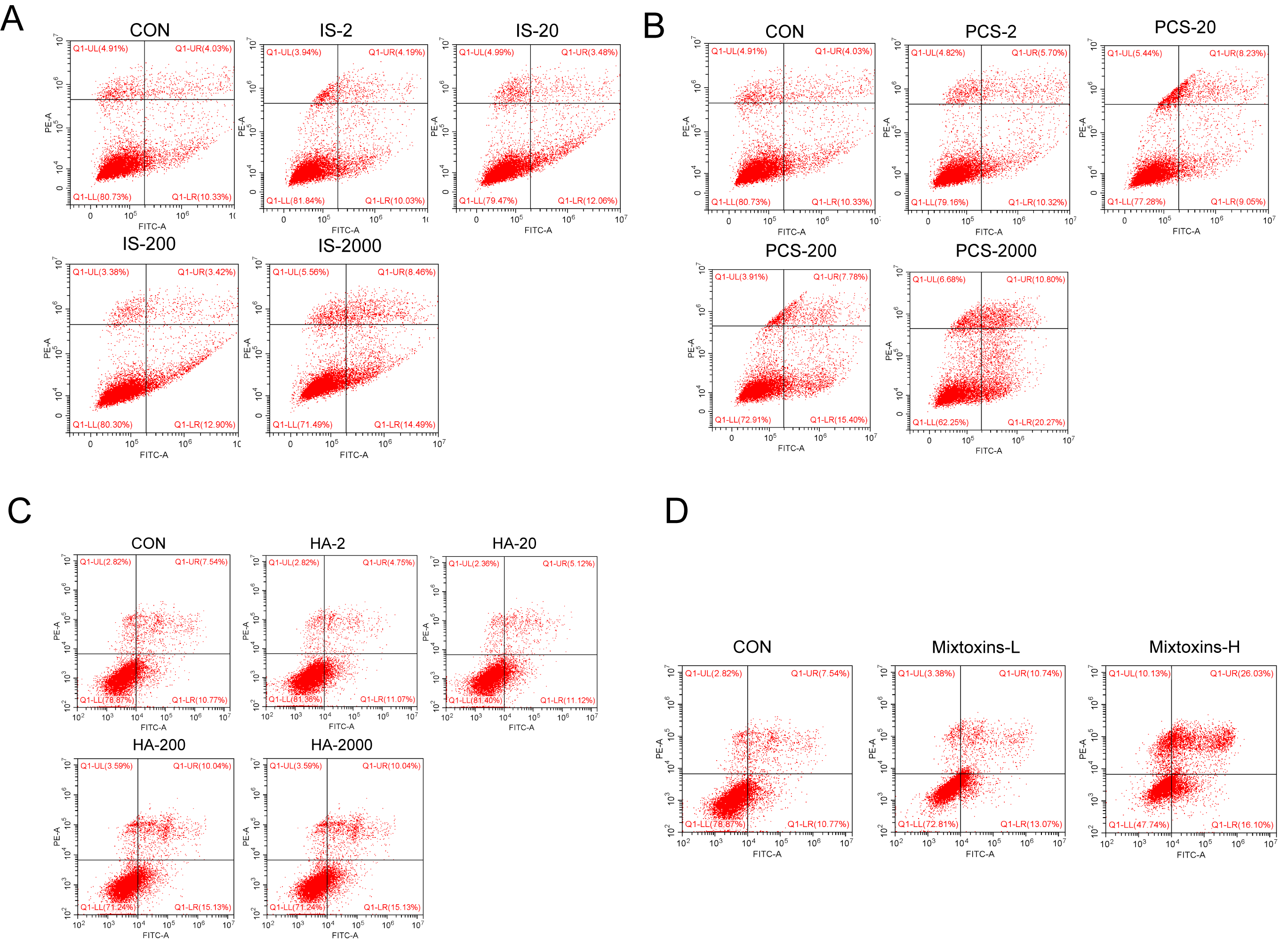

Supplement: Supplementary Figure S2 — (A) Effect of IS on THLE-2 apoptosis (n = 2-3). After 24 h treatment, flow cytometry was performed on cells stained with annexin V-FITC and PI. CON, control. IS-2, 2 μM IS treatment. IS-20, 20 μM IS treatment. IS-200, 200 μM IS treatment. IS-2000, 2000 μM IS treatment. Group settings are the same with PCS (B) and HA (C). (B) Effect of PCS on THLE-2 apoptosis (n = 2-3). (C) Effect of HA on THLE-2 apoptosis (n = 3). (D) Effects of three gut-derived PBUTs mixture on THLE-2 apoptosis (n = 3). [file Image_2.TIF]

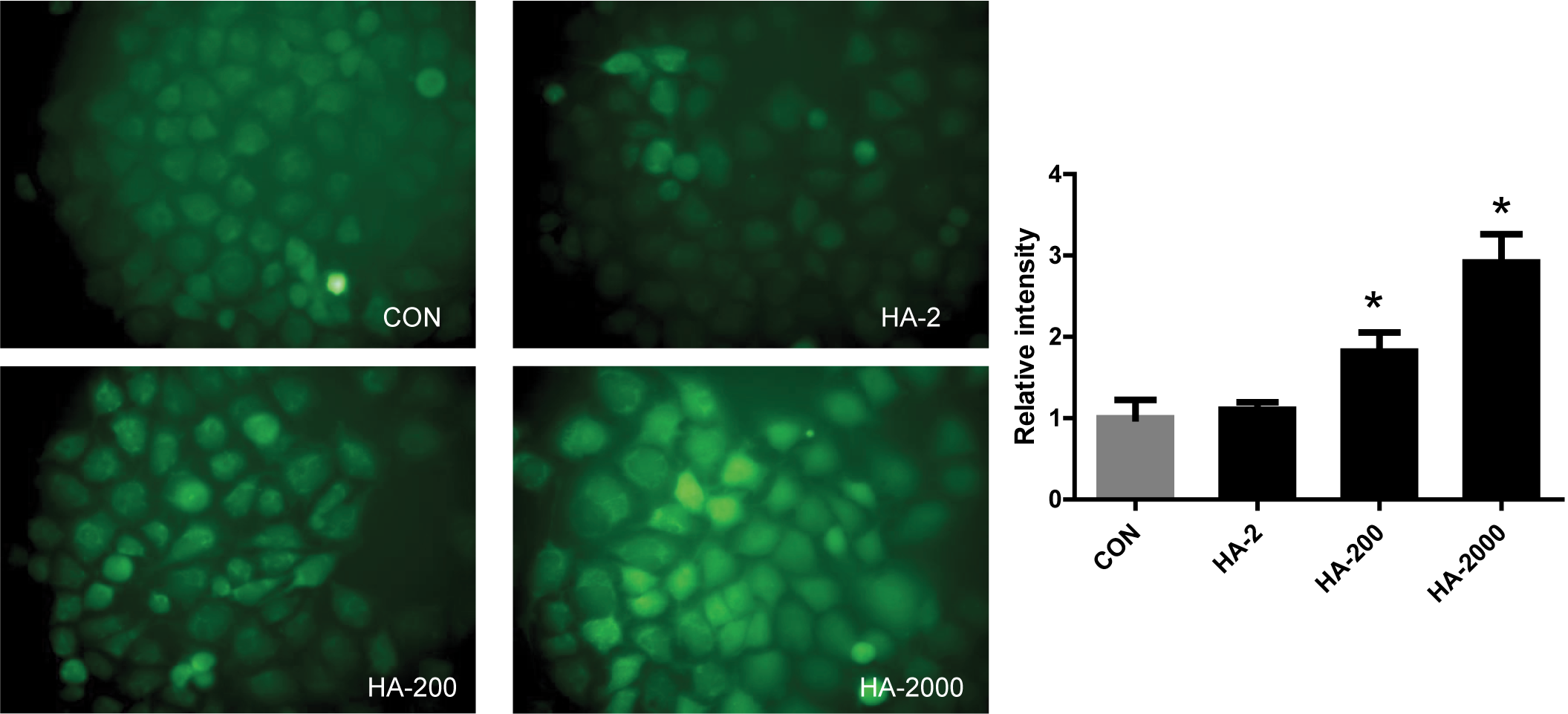

Supplement: Supplementary Figure S3 — Effects of HA-induced oxidative stress in THLE-2 cells. ROS level showed by fluorescent pictures after being treated with doses of HA, quantified by Image J software (n = 5). Data are the mean ± SD. *, P < 0. 05 vs. CON group. The significant difference was assessed using the one-way ANOVA followed by LSD posttests. [file Image_3.TIF]
